# STAT2 is involved in the pathogenesis of psoriasis by promoting CXCL11 and CCL5 production by keratinocytes

**DOI:** 10.1371/journal.pone.0176994

**Published:** 2017-05-04

**Authors:** Claus Johansen, Anne Hald Rittig, Maike Mose, Trine Bertelsen, Isabella Weimar, Jakob Nielsen, Thomas Andersen, Tue Kruse Rasmussen, Bent Deleuran, Lars Iversen

**Affiliations:** 1 Department of Dermatology, Aarhus University Hospital, Aarhus C, Denmark; 2 Department of Biomedicine, Aarhus University, Aarhus C, Denmark; San Gallicano Dermatologic Institute, ITALY

## Abstract

The JAK/STAT signaling pathway is suggested to play an important role in the pathogenesis of psoriasis, and recently JAK/STAT inhibitors have shown promising results in psoriasis treatment. The present study aimed to characterize the role of STAT2 in psoriasis. We demonstrated an increased expression of *STAT2* and an increased level of phosphorylated/activated STAT2 in lesional compared with nonlesional psoriatic skin. Gene silencing of *STAT2* by siRNA in human keratinocytes revealed that upon IFNα stimulation CXCL11 and CCL5 were the only two cytokines, among 102 analyzed, found to be regulated through a STAT2-dependent mechanism. Moreover, the regulation of CXCL11 and CCL5 depended on IRF9, but not on STAT1 and STAT6. The *CXCL11* and *CCL5* expression was increased in lesional compared with nonlesional psoriatic skin, and analysis demonstrated positive correlation between the expression of *CXCL11* and *IFNγ* and between the expression of *CCL5* and *IFNγ* in lesional psoriatic skin. In contrast, no correlation between the expression of *CXCL11* and *IL-17A* and the expression of *CCL5* and *IL-17A* in lesional psoriatic skin was found. Our data suggest that STAT2 plays a role in the psoriasis pathogenesis by regulating the expression of CXCL11 and CCL5, and thereby attracting IFNγ-producing immune cells to the skin.

## Introduction

Psoriasis is a chronic incurable systemic inflammatory disease affecting 2–3% of the total population worldwide [1]. It is characterized by hyperproliferation and an abnormal differentiation of the keratinocytes as well as infiltration of immune cells into both the dermis and the epidermis [1]. Psoriasis is known to be the result of a complex interplay between genetics, environmental triggers, and the immune system [1, 2]. Advances in our understanding of the role of intracellular signaling in psoriatic inflammation have led to the recognition that many key psoriatic cytokines converge on and initiate intracellular signaling through specific pathways [1, 3]. One such intracellular signaling pathway, which is believed to play a key role in the pathogenesis of psoriasis is the JAK/STAT signaling pathway [4–7]. Chang *et al*. showed that a small molecule inhibitor of JAK3 markedly attenuated of skin lesions following 6 weeks of treatment in the CD18 mutant PL/J mice T cell-dependent psoriasis-form model [8]. Another study demonstrated clinical activity of a topical JAK1/2 inhibitor in the treatment of psoriasis [9]. In addition, Tofacitinib, a JAK1 and JAK3 inhibitor, has shown efficacy in the treatment of moderate-to-severe chronic plaque psoriasis [10].

The STATs are ubiquitous transcription factors activated by the JAKs. They have numerous cellular functions and are involved in the regulation of several cytokine signaling pathways [11]. Seven members of the STAT family proteins are known: STAT1, STAT2, STAT3, STAT4, STAT5A, STAT5B, and STAT6 [12]. STAT2 plays a key role in type I IFN signaling by being activated and translocated to the nucleus after type I IFN receptor ligation. In the nucleus, STAT2 binds to specific promoter regions thereby activating or repressing the transcription of target genes [11]. STAT1 and STAT3 have previously been demonstrated to display an increased expression and activity in psoriatic skin [6, 7]. However, the role of STAT2 in the pathogenesis of psoriasis is unknown. The aim of the present study was therefore to characterize the role of the STAT2 signaling pathway in the pathogenesis of psoriasis.

## Materials and methods

### Biopsies

Biopsies were obtained from nonlesional and lesional skin from patients suffering from psoriasis or atopic dermatitis. Briefly, keratome and 4-mm punch biopsies were obtained from lesional and nonlesional plaque-type psoriatic skin taken from the center of a plaque from patients with moderate to severe chronic stable plaque psoriasis from either the upper or the lower extremities. Biopsies were taken as paired samples and biopsies from nonlesional psoriatic skin were taken from the same body region as biopsies from lesional psoriatic skin in a distance of at least five centimeters from a lesional plaque. The biopsies were immediately snap-frozen in liquid nitrogen. For immunofluorescence analysis, 4-mm punch biopsies were taken, and embedded in paraffin. The study was approved by the Regional Ethical Committee of Region Midtjylland, Denmark (M-20090102), and was conducted in compliance with the Declaration of Helsinki. Signed informed consent was obtained from each patient.

### Cell cultures

Normal adult human keratinocytes were obtained by trypsinization of skin samples from patients undergoing plastic surgery as previously described [13]. Second-passage keratinocytes were grown in keratinocyte serum-free medium (KSFM) (Gibco, Invitrogen, Carlsbad, CA). 24 hours before stimulation with IFNα (1000 U/ml, cat. no. 11200–1, R&D Systems, Oxon, UK), the medium was changed to keratinocyte basal medium (KBM, the same as KSFM but without growth factors) in which the cells were stimulated. Cells were grown at 37°C and 5% CO_2_ in an incubator. The Regional Ethical Committee of Region Midtjylland, Denmark approved the experiments with cultured human keratinocytes (M-20110027).

### Isolation of PBMCs

Fresh whole blood were taken from psoriatic patients and added to tubes containing EDTA. The EDTA blood was mixed 1:1 with Hanks buffered saline and separated by centrifugation using Lymphoprep (Axis-Shield, Cambridgeshire, UK). The PBMC layer was then isolated and washed three times in Hanks buffered saline before being resuspended in RPMI-1640 medium supplemented with 2% FCS, 100 U/ml penicillin, 100 μg/ml streptomycin and 5 μg/ml gentamycin.

### Chemotaxis assay

Chemotaxis assays were performed using a 24-well plate based assay (cat no. CBA-125; Cell Biolabs, Inc., San Diego, CA). Briefly, isolated PBMCs from psoriatic patients were resuspended in RPMI-1640 medium containing 2% FCS, and 100 μl medium (5x10^5^ cells) were loaded on the upper chamber containing a 5-μm pore-size filter. Wells (lower chamber) were added RPMI-1640 medium + 2% FCS with or without 10 nM recombinant CXCL11 or CCL20. Then the plate was incubated at 37°C and 5% CO_2_ in an incubator for 20 hours. Cells migrating to the lower chamber were analyzed by flow cytometry.

### Flow cytometry

For intracellular flow cytometry, cells were stimulated for 4 hours with 50 ng/mL phorbol 12-myristate 13-acetate and 1 μg/mL ionomycin in the presence of 10 μg/mL Brefeldin A (all Sigma-Aldrich, St. Louis, MO). Cells were then stained with anti-CD4 PE-Cy7 (cat. no. 557852; BD Biosciences, San Jose, CA) and Live/Dead Near-IR Dead Cell Stain (cat. no. L10119; Invitrogen, Carlsbad, CA). Cells were permeabilized using 0.3% saponin (Sigma-Aldrich) in PBS/BSA/Azide. All samples were blocked with 10% heat-inactivated normal mouse serum (in-house production) after permeabilization before being stained with anti-interferon-γ PE antibody (cat. no. 562016; BD Biosciences). All antibodies used were titrated to optimal working concentration. Cells were first gated for viability using the Live/Dead stain followed by doublet exclusion using a side scatter height vs. area plot and finally gated for CD4 expression and analyzed for IFNγ expression using Cytobank [14].

### Immunofluorescence analysis

4-μm sections of paraffin-embedded tissue samples from nonlesional and lesional psoriatic skin were deparaffinized and heated at 95°C for 15 minutes in a Tris/EGTA (pH 9.0) buffer for antigen unmasking. The samples were then blocked for 30 minutes with Image-iT^™^ FX Signal Enhancer (Life Technologies, Austin, TX) before incubation with anti-STAT2 antibody (1:50) (cat. no. sc-476; Santa Cruz Biotechnology, Santa Cruz, CA) in blocking buffer overnight. The samples were washed and incubated with AlexaFluor^®^ 488 secondary antibody (Life Technologies) for 1 hour. Finally, the samples were washed and nuclear staining was performed by embedding samples in Prolong Gold antifade reagent with DAPI (Life Technologies). For analysis of STAT2 expression, cultured human keratinocytes were grown on chamber slides (BD Biosciences, Bedford, MA). Cells were fixed in ice-cold methanol for 5 minutes at room temperature before heated at 95°C for 8 minutes in a Tris/EGTA (pH 9.0) buffer for antigen unmasking. Then cells were blocked, incubated with primary and secondary antibodies, and stained with DAPI as described above.

Samples were evaluated by epifluorescence microscopy. As negative control, sections were incubated with blocking buffer without primary antibody, and as isotype control with normal rabbit IgG (cat no. sc-2027; Santa Cruz Biotechnology) instead of primary antibody.

### Quantitative PCR

For quantitative PCR, Taqman Reverse Transcription reagents, primers and probes were purchased from Life Technologies. *STAT2*, *CXCL11*, *CCL5*, *IL-17A*, and *IFNγ* mRNA expression was analyzed using Taqman 20X Assays-On-Demand expression assay mix (assay ID: Hs01013123_m1, Hs04187682_g1, Hs00982282_m1, Hs00174383_m1 and Hs00989291_m1, respectively). The probe was a FAM-labeled MGB probe with a non-fluorescent quencher. As housekeeping gene, we used *RPLP0*. *RPLP0* mRNA expression was determined by using Taqman 20X Assays-On-Demand expression assay mix (assay ID: Hs99999902_m1). The probe was a FAM-labeled MGB probe with a non-fluorescent quencher.

PCR mastermix was Platium^®^ qPCR Supermix-UDG (Life Technologies). Each gene was analyzed in triplicates. The real-time PCR machine was a Rotorgene-3000 (Corbett Research, Sydney, Australia). Reactions were run as singleplex. Relative gene expression levels were determined by using the relative standard curve method as outlined in User Bulletin #2 (ABI Prism 7700 sequencing detection system, Life Technologies). Briefly, a standard curve for each gene was made of 4-fold serial dilutions of total RNA from punch biopsies from the skin of psoriatic patients. The curve was then used to calculate relative amounts of target mRNA in the samples.

### Western blot analysis

Keratome biopsies were homogenized in a cell lysis buffer (20 mM Tris-base (pH 7.5), 150 mM NaCl, 1 mM EDTA, 1 mM EGTA, 1% Triton X-100, 2.5 mM sodidum pyrophosphate, 1 mM β-glycerolphosphate, 1mM Na_3_VO_4_, 1 μg/ml leupeptin, and 1 mM PMSF) as previously described [15]. The samples were then centrifuged at 10,000 x g for 10 minutes at 4°C, after which the supernatant constituted the cell lysate. The protein extracts from cultured human keratinocytes were isolated as previously described [16].

Equal protein amounts were separated by SDS-PAGE and blotted onto nitrocellulose membranes. Membranes were incubated with anti-phospho-STAT2(Tyr690), anti-STAT2, anti-STAT1 (cat. no. 4441, 4594, and 9172, respectively; Cell Signaling Technology, Danvers, MA), anti-IRF9 (cat. no. AF5629, R&D Systems, Oxon, UK), or anti-β-actin (cat. no. A-1978; Sigma-Aldrich, St. Louis, MO). The antibodies were detected with anti-rabbit IgG-HRP (cat. no. 7074; Cell Signaling Technology, Danvers, MA) or with anti-sheep IgG-HRP (cat. no. P0163; Dako, Glostrup, Denmark) in a standard ECL reaction (Amersham Biosciences, Piscataway, NJ) according to the manufacturer’s instructions. Densitometric analysis of the band and background intensities was conducted using Kodak 1D Image analysis software. Results were normalized to β-actin levels.

### ELISA

CXCL11, IL-1α, IL-1ra, CCL5, MCP-1, IL-8, CXCL1, and ENA-78 protein levels in cultured normal human keratinocytes were measured using DuoSet ELISA kits from R&D Systems, Oxon, UK (cat. no.: DY672, DY200, DY280, DY278, DY279, DY208, DY275, and DY254, respectively). The ELISA was carried out according to the manufacturer’s protocol. The final result was determined by an ELISA reader (Laboratory systems iEMS Reader MF, Copenhagen, Denmark) at 450 nm. All measurements were performed in doublets.

### siRNA transfection

Cultured human keratinocytes were grown to 60–70% confluency. Before transfection, the cells were changed to medium without growth factors (KBM). siRNA directed against STAT1, STAT2, STAT6, or IRF9 (cat. no. L-003543-00-0005, L-013497-00-0005, L-006690-00-0005 and L-020858-00-0005, respectively; Dharmacon, Lafayette, CO) was preincubated with Dharmafect-2 transfection reagent (Dharmacon) in KBM for 20 minutes. The formed siRNA/transfection reagent complexes were added to the cells to a final concentration of 10 nM. As negative controls, cells were transfected with siControl nontargeting pool siRNA (cat. no. D-001810-10-05, Dharmacon) or the transfection reagent alone (mock). Five hours after transfection, the medium was changed to keratinocyte growth medium (growth factors included). 24 hours before stimulation, the medium was changed to KBM.

### Statistical analysis

**Fig 1:**
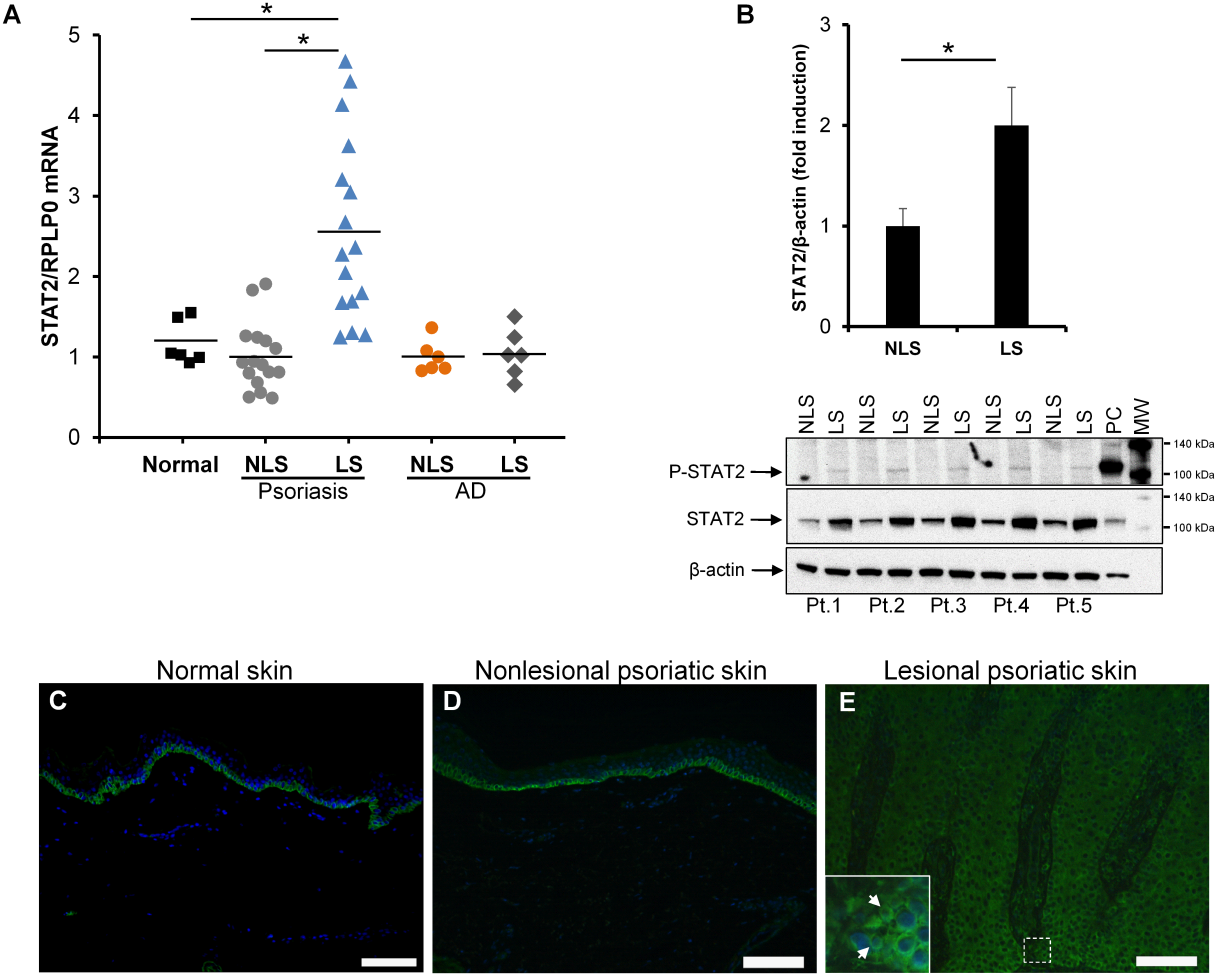
STAT2 expression is elevated in psoriatic skin. (A) *STAT2* mRNA expression was examined by qPCR in biopsies obtained from normal healthy volunteers as well as from paired punch biopsies obtained from nonlesional (NLS) and lesional (LS) skin from patients suffering from psoriasis or atopic dermatitis (AD). The mRNA expression of *RPLP0* was used for normalization. Biopsies from 6 healthy volunteers, 16 psoriatic patients and 6 atopic dermatitis patients were examined. The results are presented as dot plots with the horizontal line expressing the mean value. All measurements were performed in triplicates. (B) Whole cell protein extracts were prepared from paired keratome biopsies taken from nonlesional and lesional psoriatic skin from five psoriatic patients. Phosphorylated STAT2 as well as total STAT2 protein was analyzed by western blotting. Equal protein loading was assessed by detecting the protein level of β-actin. Protein extract from keratinocytes stimulated with IFNα for 1 hour was included as a positive control (PC). MW; molecular weight marker. Densitometic analysis of the band intensity was carried out and values were normalized to β-actin. *P < 0.01. (C-E) Immunofluorescence analysis was performed on paraffin-embedded punch biopsies from (C) normal skin as well as (D) nonlesional and (E) lesional psoriatic skin. Nuclear staining was performed using 4’, 6-diamidine-2’-phenylindole dihydrochloride (DAPI). Green color (Alexa Fluor 488) represents STAT2 protein. Three sets of biopsies from three different patients were investigated. Arrows show nuclear staining. Scale bar = 100 μm.

Statistical differences among the experimental groups were analyzed by use of one way anova after testing for normality. In Fig 1B, a Students *t*-test was used. A probability of *P* < 0.05 was regarded as statistically significant.

## Results

### STAT2 expression and phosphorylation are increased in lesional psoriatic skin

In order to examine *STAT2* mRNA expression in psoriatic skin, RNA was isolated from punch biopsies obtained from both lesional and nonlesional skin from 16 psoriatic patients and 6 healthy controls. Using qPCR analysis, we demonstrated a significantly elevated *STAT2* mRNA expression in lesional psoriatic skin compared with nonlesional psoriatic skin and normal healthy controls, with a mean approximately 2.5-fold increase. No difference in *STAT2* mRNA expression was observed between nonlesional psoriatic skin and normal healthy skin (Fig 1A). In order to study if the observed increase in *STAT2* expression was specific to psoriasis or simply due to increased inflammation in the skin we also investigated the *STAT2* expression in the inflammatory skin disease atopic dermatitis. We found no changes in the *STAT2* expression between lesional and nonlesional atopic dermatitis skin. Moreover, the *STAT2* expression in skin from patients with atopic dermatitis was comparable to the expression level seen in normal healthy controls (Fig 1A). Activated STAT2 is known to be implicated in the regulation of genes involved in numerous cellular processes including inflammation [17]. To study the activity/phosphorylation of STAT2 in psoriatic skin, protein extracts from paired keratome biopsies were isolated. Western blotting analysis using an antibody directed against phosphorylated STAT2 revealed that the level of phosphorylated/activated STAT2 was clearly increased in lesional psoriatic skin compared with nonlesional psoriatic skin (Fig 1B). The full-length western blots for phosphorylated STAT2, total STAT2 and β-actin in psoriatic skin are shown in figure S1 (S1 Fig). The phosphorylation level of STAT2 was also examined in lesional and nonlesional skin from patients with atopic dermatitis. In contrast to psoriatic skin, no alteration in the STAT2 phosphorylation level between lesional and nonlesional skin from patients with atopic dermatitis were observed (S2 Fig). Together, these data indicate that STAT2 upregulation and activation in psoriatic skin is not simply due to inflammation in the skin, but seems to be more specific to psoriasis. The increased mRNA expression of *STAT2* in lesional psoriatic skin was paralleled by an increased STAT2 protein level as demonstrated by western blotting, showing a mean increase of ~2.1-fold (Fig 1B).

Because *STAT2* demonstrated an increased expression in psoriatic skin, we next examined the STAT2 localization in lesional and nonlesional psoriatic skin as well as in normal healthy skin. Immunofluorescence analysis was conducted using 4-μm sections of paraffin-embedded punch biopsies from normal healthy skin and from paired biopsies from lesional and nonlesional psoriatic skin. In normal healthy skin and nonlesional psoriatic skin, positive STAT2-stained cells were observed exclusively in the basal layer of the epidermis (Fig 1C and 1D). In contrast, positive-stained cells scattered throughout the entire epidermis were observed in lesional psoriatic skin (Fig 1E). As a positive control, keratinocytes were stimulated with or without IFNα after which STAT2 was examined by immunocytochemistry. As expected, nuclear localization of STAT2 was observed in IFNα-treated cells (S3 Fig). Together, these data suggest that STAT2 may play a role in the pathogenesis of psoriasis.

### CXCL11 and CCL5 expression are induced by IFNα through a STAT2-dependent mechanism in cultured normal human keratinocytes

**Fig 2:**
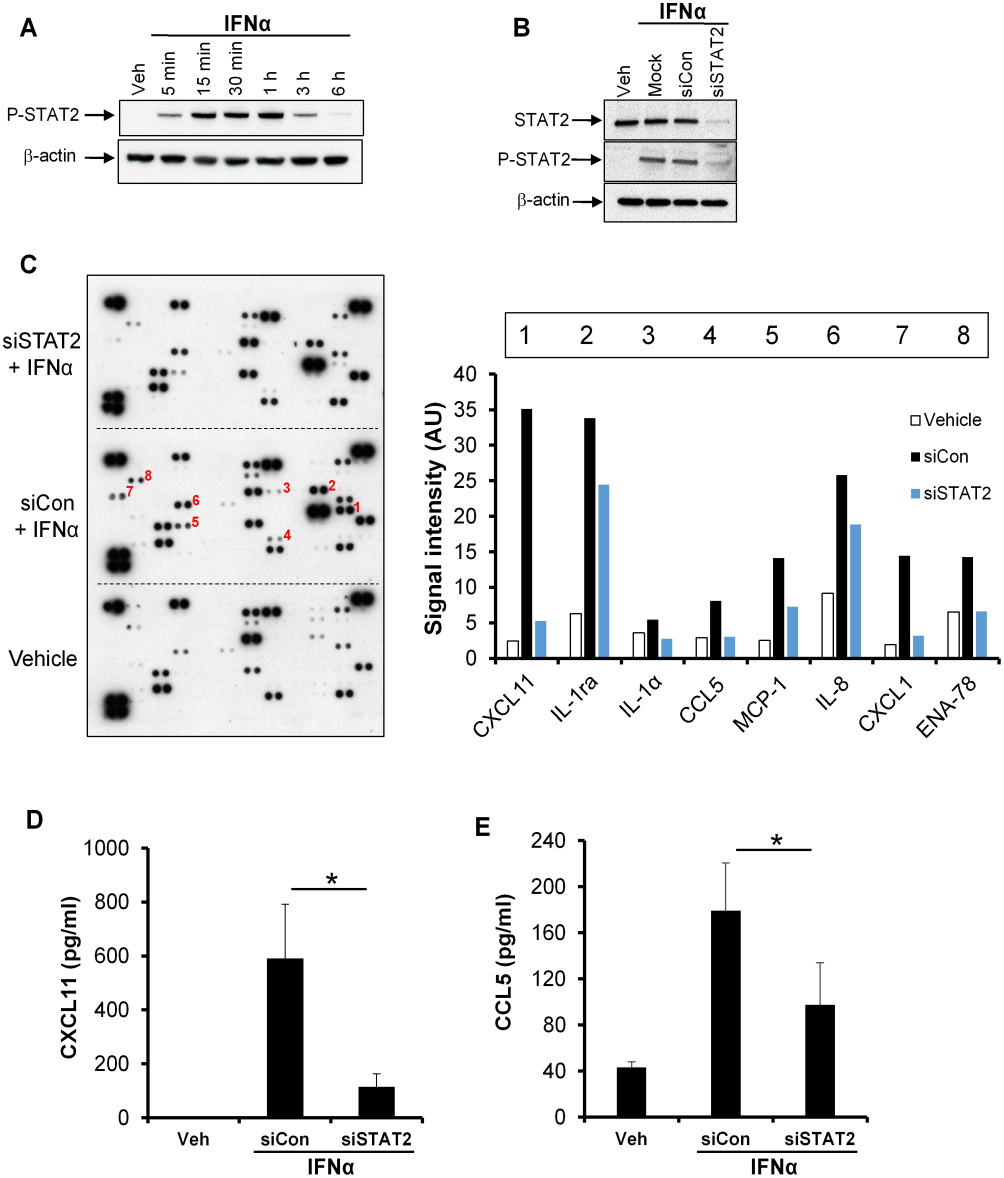
STAT2-mediated regulation of CXCL11 and CCL5 expression. (A) Cultured human keratinocytes were stimulated with IFNα (1000 U/ml) for the indicated time points, and the phosphorylation level of STAT2 analyzed by western blotting (n = 3). (B) Keratinocytes were transfected with STAT2 siRNA (siSTAT2), control siRNA (siCon) or transfection reagent alone (Mock) before being stimulated with IFNα (1000 U/ml) for 24 hours. Protein extracts were isolated from the cells and STAT2 and P-STAT2 expression analyzed by western blotting (n = 3). β-actin was used as a loading control. (C) Human keratinocytes were transfected with STAT2 siRNA (siSTAT2) or control siRNA (siCon) before stimulation with IFNα (1000 U/ml) for 24 hours. Then the cell culture medium was screened for 102 proinflammatory cytokines and chemokines using a proteome profile array. Numbers on the membrane marks the following targets: 1 (CXCL11), 2 (IL-1ra), 3 (IL-1α), 4 (CCL5), 5 (MCP-1), 6 (IL-8), 7 (CXCL1) and 8 (ENA-78). (D, E) Human keratinocytes were stimulated as in (C) and the expression level of CXCL11 and CCL5 analyzed by ELISA (n = 3). Results are expressed as mean ± standard deviation. *P < 0.05.

IFNα is known to be an activator of STAT2 [18]. To further characterize STAT2, we next stimulated cultured human keratinocytes with IFNα (1000 U/ml) at different time points. IFNα induced STAT2 phosphorylation in human keratinocytes in a time-dependent manner with a maximum increase after 1 hour of stimulation. Six hours after IFNα stimulation, the phosphorylation level of STAT2 had almost returned to the level seen in vehicle-treated cells (Fig 2A). To analyze the role of STAT2 in IFNα-induced gene expression, we used small interfering RNA (siRNA) to knockdown STAT2. Transfection of cultured human keratinocytes with specific STAT2 siRNA clearly reduced the protein level of STAT2 by approximately 75% compared with IFNα-stimulated cells transfected with control siRNA (Fig 2B). In addition, STAT2 knockdown also resulted in a reduced level of the phosphorylated form of STAT2 in IFNα-stimulated cells compared with control siRNA transfected cells (Fig 2B). Using a proteome array, we next analyzed the effect of STAT2 knockdown on the IFNα-induced expression level of 102 different inflammatory cytokines. Human keratinocytes were transfected with STAT2 siRNA or non-targeting control siRNA before being stimulated with IFNα for 24 hours. Based on this screening assay, eight cytokines (CXCL11, IL-1ra, IL-1α, CCL5, MCP-1, IL-8, CXCL1 and ENA-78) were found, not only to be induced by IFNα stimulation, but also to have a reduced expression level when STAT2 was knocked down by siRNA (Fig 2C). Thereafter ELISA was conducted to further characterize the role of STAT2 in the IFNα-induced expression of these eight cytokines. The protein level of both CXCL11 and CCL5 was significantly reduced in cells transfected with STAT2 siRNA before stimulation with IFNα for 24 hours (Fig 2D and 2E). Although the proteome screening array suggested that STAT2 also played a role in the regulation of IL-1ra, IL-1α, MCP-1, IL-8, CXCL1, and ENA-78, this could not be confirmed by ELISA which showed that STAT2 knockdown had no effect on the IFNα-induced expression level of these cytokines (S4 Fig).

### IFNα-induced CXCL11 and CCL5 expression are regulated through a STAT2- and IRF9-dependent, but STAT1- and STAT6-independent mechanism

**Fig 3:**
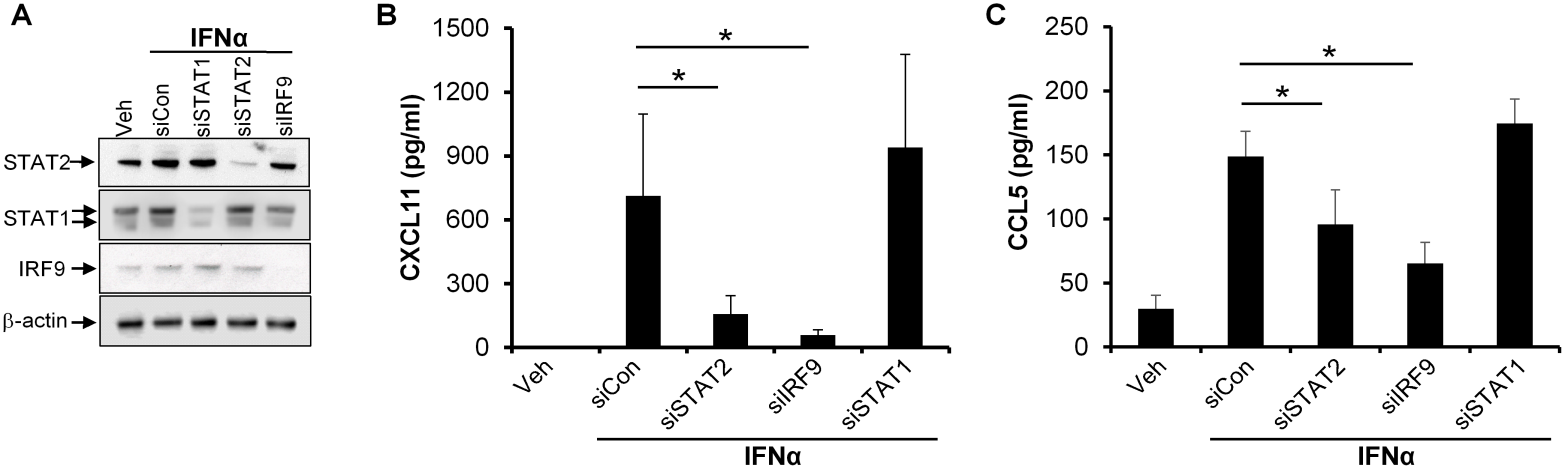
IFNα-induced CXCL11 and CCL5 expression involves STAT2 and IRF9, but not STAT1. Cultured human keratinocytes were transfected with siRNA directed against STAT2 (siSTAT2), STAT1 (siSTAT1), IRF9 (siIRF9), or control siRNA (siCon) before IFNα (1000 U/ml) stimulation for 24 hours. (A) Protein extracts were isolated from the cells and the protein level of STAT2, STAT1, and IRF9 examined by western blotting. β-actin was used as a loading control. One representative gel out of three is shown. (B, C) The protein level of (B) CXCL11 and (C) CCL5 in the cell culture medium was analyzed by ELISA (n = 3). Results are expressed as mean ± standard deviation. *P < 0.05.

Because CXCL11 and CCL5 were the only two cytokines, among 102 analyzed, found to be regulated through a STAT2-dependent mechanism upon IFNα stimulation, we next characterized the molecular mechanism involved. Upon IFNα stimulation, STAT2 has been described to form a complex consisting of STAT1 and IRF9 which binds to DNA and thereby regulates gene transcription [19]. In order to examine whether STAT1 and IRF9 were involved in the IFNα-induced regulation of CXCL11 and CCL5, we used siRNA to knock down STAT1 and IRF9. Transfection of cultured human keratinocytes with STAT1 siRNA or IRF9 siRNA reduced the protein level of STAT1 and IRF9, respectively, in IFNα-stimulated cells compared with cells transfected with nontargeting control siRNA (Fig 3A). In the same cells, we analyzed the level of CXCL11 and CCL5 by ELISA. Interestingly, siRNA-mediated knockdown of IRF9 significantly reduced IFNα-induced CXCL11 and CCL5 expression compared with control siRNA-transfected cells (Fig 3B and 3C). In contrast, knockdown of STAT1 had no effect on the IFNα-induced expression of CXCL11 and CCL5, demonstrating that IFNα regulates the expression of CXCL11 and CCL5 by a STAT2- and IRF9-dependent, but STAT1-independent mechanism. Because STAT2 and STAT6 have also been demonstrated to form complexes upon IFNα-mediated signaling [19, 20], we investigated whether STAT6 was involved in the regulation of CXCL11 and CCL5. Although transfection of cells with specific siRNA directed against STAT6 led to a clear knockdown of STAT6 in IFNα-stimulated cells, silencing of STAT6 had no effect on the IFNα-induced CXCL11 and CCL5 protein level (S5 Fig). This demonstrates that IFNα regulates the expression of CXCL11 and CCL5 by a mechanism that is independent of STAT6.

### *CXCL11* and *CCL5* expression are increased in lesional psoriatic skin

**Fig 4:**
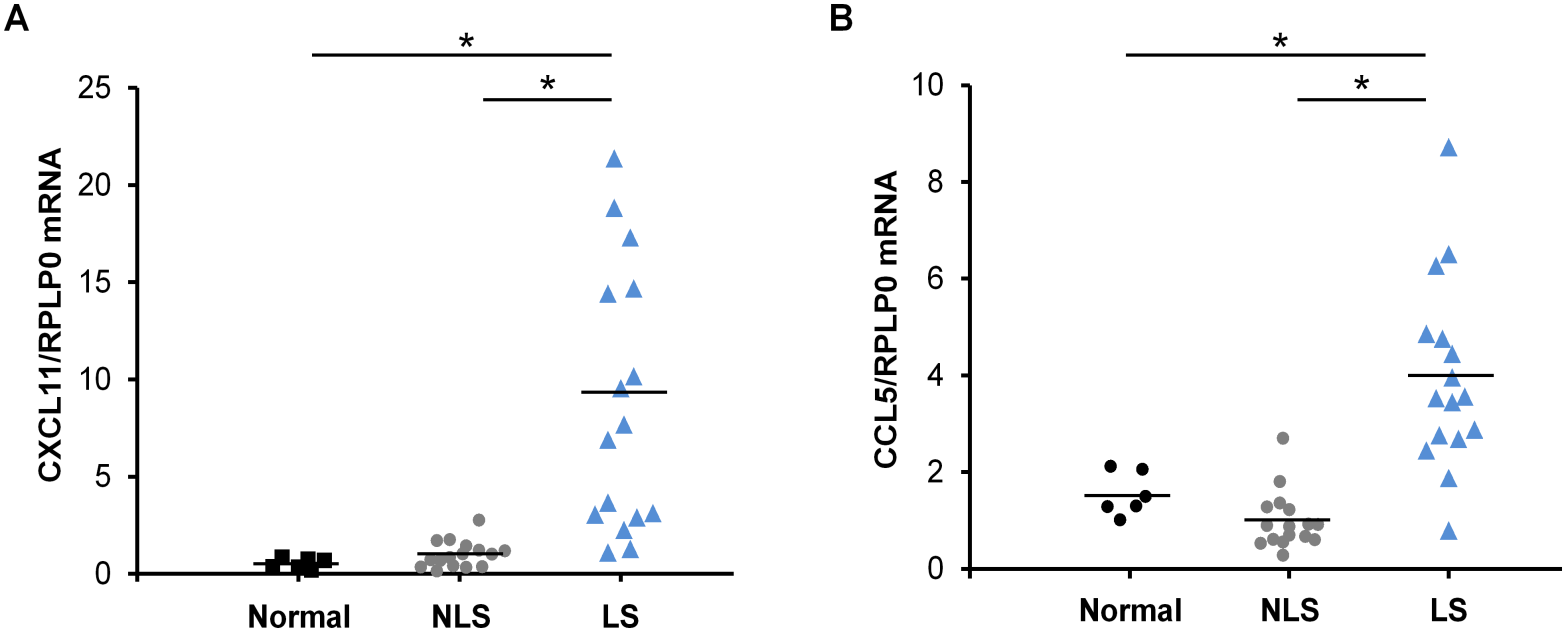
Increased mRNA expression of *CXCL11* and *CCL5* in lesional psoriatic skin. Total RNA was isolated from biopsies obtained from normal healthy volunteers as well as lesional and nonlesional psoriatic skin. mRNA expression of (A) *CXCL11* and (B) *CCL5* was analyzed by qPCR. *RPLP0* mRNA expression was used for normalization. Scatterplot shows the result from 6 healthy volunteers and 16 psoriatic patients. *P < 0.01.

As the phosphorylation/activity of STAT2 was increased in lesional psoriatic skin, and the expression of CXCL11 and CCL5 was found to be regulated through a STAT2-dependent mechanism *in vitro*, we next examined the expression level of *CXCL11* and *CCL5* in skin biopsies from 16 patients with psoriasis and from 6 healthy controls. We demonstrated that the mRNA expression of *CXCL11* and *CCL5* was significantly increased in lesional psoriatic skin where a ~9-fold and a ~4-fold increase were observed, respectively, compared with nonlesional psoriatic skin from the same patient (Fig 4A and 4B). We found no difference in the expression level of *CXCL11* and *CCL5* between nonlesional psoriatic skin and skin from normal healthy controls (Fig 4A and 4B).

### *CXCL11* and *CCL5* expression are positively correlated with *IFNγ* expression in psoriatic skin

**Fig 5:**
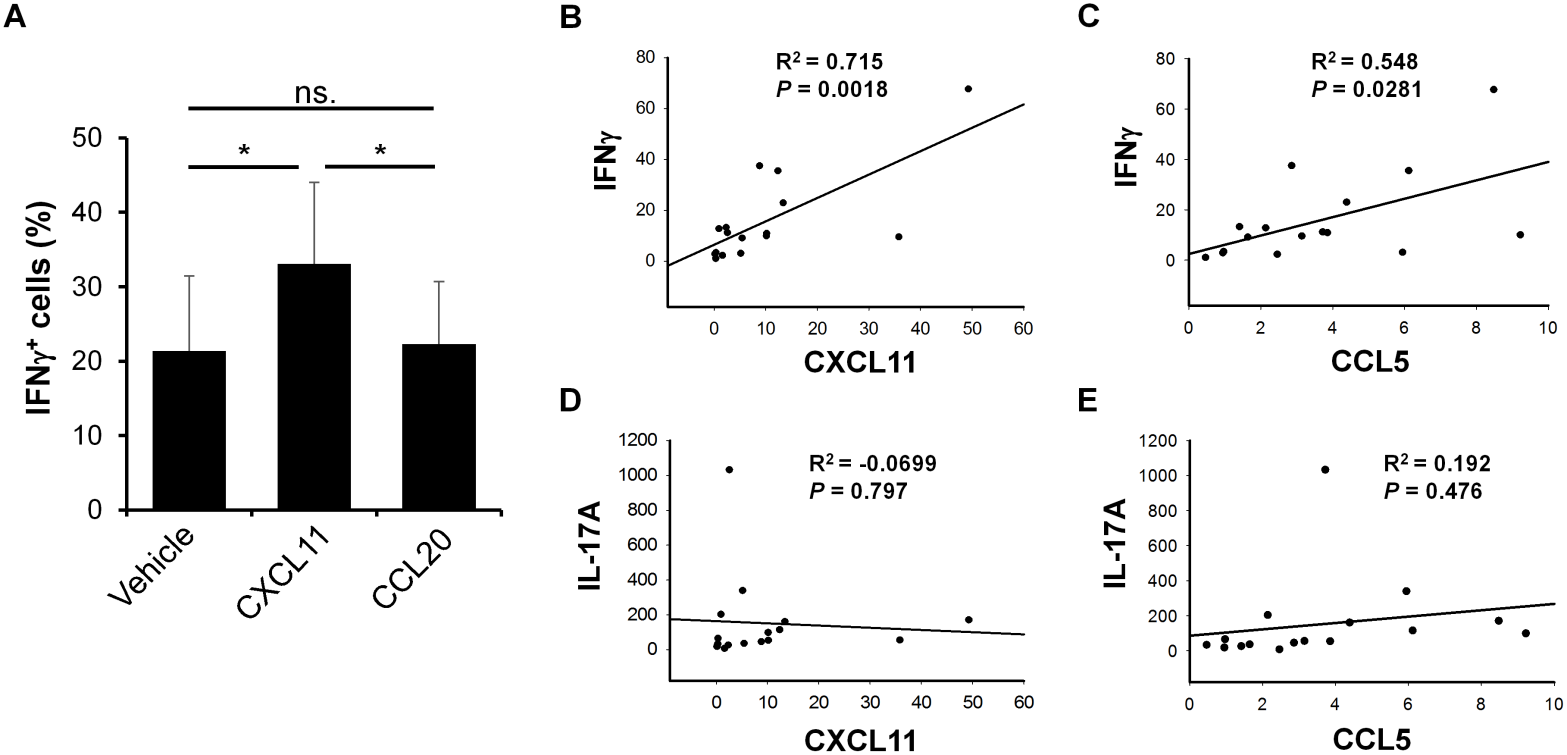
The expression of *CXCL11* and *CCL5* is positively correlated with IFNγ expression in lesional psoriatic skin. (A) Chemotaxis assays were performed using a 24-well plate-based assay. PBMCs were isolated from blood taken from psoriatic patients and loaded on the upper chamber containing a 5-μm pore-size filter. Wells (lower chamber) were added vehicle or 10 nM recombinant CXCL11 or CCL20 and incubated for 20 hours. Cells migrating to the lower chamber were analyzed by flow cytometry (n = 9). Results are expressed as mean ± standard deviation. *P < 0.01. (B-E) RNA from punch biopsies taken from 16 psoriatic patients was isolated and the mRNA expression of *CXCL11*, *CCL5*, *IFNγ*, and *IL-17A* analyzed by qPCR. The correlation between the expression levels of these cytokines was analyzed as indicated in the figure.

Because CXCL11 is known to be a strong chemoattractant for Th1 cells by binding to CXCR3 [21], we next analyzed the ability of CXCL11 to attract Th1 cells from PBMCs isolated from psoriatic patients. By conducting chemotaxis assays, we demonstrated a significantly higher percentage of IFNγ-producing cells when CXCL11 was used as a chemoattractant compared with vehicle. In contrast, when CCL20 was used as a chemoattractant, no difference in the number of IFNγ-producing cells was observed compared with vehicle (Fig 5A). Furthermore, we demonstrated a positive correlation between the expression of *CXCL11* and *IFNγ* and between the expression of *CCL5* and *IFNγ* in lesional psoriatic skin (Fig 5B and 5C). In contrast to this, no correlation was observed between the expression of *CXCL11* and *IL-17A* or the expression of *CCL5* and *IL-17A* in lesional psoriatic skin (Fig 5D and 5E). These data suggest that in psoriasis, upregulated CXCL11 and CCL5 are involved in the recruitment of IFNγ-producing Th1 cells to the skin.

## Discussion

The JAK/STAT pathways are intracellular signaling pathways, which are believed to play a key role in numerous inflammatory responses [22]. Recently, several small molecule inhibitors directed against the JAK/STAT signaling pathway have shown promising results in the treatment of inflammatory diseases, including psoriasis [8, 9, 23–25]. The role of the STAT proteins in the pathogenesis of psoriasis as well as the mechanisms involved in the regulation and activation of the different STAT proteins are, however, not fully understood.

We here present three essential and novel findings, adding new knowledge to the role of STAT2 in the pathogenesis of psoriasis. First, we demonstrated an increased expression of *STAT2* as well as an elevated phosphorylation/activation of STAT2 in lesional psoriatic skin compared with nonlesional psoriatic skin. Second, in cultured human keratinocytes, CXCL11 and CCL5 were identified as the only two cytokines regulated by a STAT2-dependent mechanism upon IFNα stimulation among 102 cytokines investigated. Third, we demonstrated a positive correlation between *CXCL11* and *IFNγ* expression as well as *CCL5* and *IFNγ* expression in lesional psoriatic skin. In contrast, no correlation was demonstrated between *CXCL11* and *IL-17A* expression and *CCL5* and *IL-17A* expression in lesional psoriatic skin.

Psoriasis is a chronic inflammatory skin disease characterized by increased infiltration of IFNγ-producing Th1 cells as well as IL-17A-producing Th17 cells in the lesional plaques [26]. A model of psoriasis initiation involving the antimicrobial peptide LL-37 and IFNα has been suggested [27, 28]. Keratinocytes produce LL-37 in response to skin injury. LL-37 in complex with DNA can activate plasmacytoid dendritic cells (DCs) through binding to Toll-like receptor (TLR) 9 resulting in IFNα production, activation of myeloid DCs and subsequently development of a psoriatic plaque [27, 28]. Our data showed an increased expression and phosphorylation/activation of STAT2 in psoriatic skin as well as a STAT2-specific regulation of *CXCL11* and *CCL5* expression in human keratinocytes upon IFNα stimulation. Interestingly, when examining STAT2 in the inflammatory skin disease atopic dermatitis, no increase in STAT2 activation/phosphorylation was detected, suggesting that STAT2 activation is specific to psoriasis and not just a consequence of skin inflammation in general. It is therefore possible that STAT2 plays an important role in the migration of immune cells in psoriasis. CXCL11 and CCL5 are two chemokines functioning as chemoattractants for primarily IFNγ-producing Th1 cells, but also for other immune cells [21, 29]. Here, we demonstrated an increased expression of both *CXCL11* and *CCL5* in lesional psoriatic skin, which was found to be positively correlated with the expression of *IFNγ*, a specific Th1-produced cytokine. It is therefore tempting to speculate that STAT2 plays a role in the pathogenesis of psoriasis by specifically recruiting Th1 cells to the inflammatory site through its regulation of CXCL11 and CCL5. The specificity for Th1 cells was also supported by the fact that no correlation was observed between the expression of these two chemokines and *IL-17A* in lesional psoriatic skin.

Previous studies have shown both an increased expression of *STAT1* in psoriatic skin and an increased STAT1 activity as measured by elevated phosphorylation levels of STAT1(Tyr701) and STAT1(Ser727), which suggests that STAT1 plays a role in the pathogenesis of psoriasis [5, 7, 30]. Furthermore, STAT1 is known to play an essential role in the regulation of IFNα-induced genes by forming a heterotrimer with STAT2 and IRF9, a transcription factor complex known as IFN-stimulated gene factor 3 (ISGF3). ISGF3 binds to the promoter region of specific target genes regulating their transcription [31]. While the activation of the ISGF3 complex has been widely suggested as a hallmark of IFNα signaling, accumulating data point in the direction of a far more multifaceted activation beyond ISGF3 [19, 20, 32]. Interestingly, in the present study, we demonstrated that IFNα induced the expression of CXCL11 and CCL5, and that this induction was mediated by a STAT1-independent, but STAT2- and IRF9-dependent mechanism. Moreover, although it has been demonstrated that STAT6 is capable also of forming a complex with STAT2 and IRF9 upon IFNα signaling [20], we found that STAT6 was not involved in the regulation of CXCL11 and CCL5 expression. Thus, these data suggest that STAT2 is the main transcription factor for IFNα-induced transcription of *CXCL11* and *CCL5* in human keratinocytes.

Taken together, our study sheds light on STAT2 expression and activation in psoriasis and the underlying molecular mechanism involved. Moreover, it offers evidence that STAT2 may be a new target for modulating Th1 cell migration in psoriasis.

## Supporting information

S1 Fig Full-length western blots showing phospho-STAT2, total STAT2 and β-actin in psoriatic skin. Full-length western blots of the data presented in Fig 1B.
(PDF)

S2 Fig Analysis of phospho-STAT2 in skin from patients with atopic dermatitis. Whole cell protein extracts were prepared from paired keratome biopsies taken from nonlesional (NLS) and lesional (LS) skin from five patients with atopic dermatitis. Phosphorylated STAT2 was analyzed by western blotting. Equal protein loading was assessed by detecting the protein level of β-actin. Protein extract from keratinocytes stimulated with IFNα for 1 hour was included as a positive control (PC). MW; molecular weight marker.
(PDF)

S3 Fig Staining of STAT2 in cultured human keratinocytes. Human keratinocytes were stimulated with or without IFNα (1000 U/ml) for 1 hour. Then the cells were fixed in methanol before immunostaining was performed using an antibody directed against STAT2. Nuclear staining was performed using 4’, 6-diamidine-2’-phenylindole dihydrochloride (DAPI). Green color (Alexa Fluor 488) represents STAT2 protein. Scale bar = 50 μm.
(PDF)

S4 Fig Effect of STAT2 knockdown on IFNα-induced cytokine expression. Cultured human keratinocytes were transfected with STAT2 siRNA (siSTAT2) or control siRNA(siCon) before IFNα stimulation for 24 hours. Then the cell culture medium was analyzed for the protein level of (A) IL-1ra, (B) IL-1α, (C) MCP-1, (D) IL-8, (E) CXCL1, and (F) ENA-78 by ELISA (n = 3). Results are expressed as mean ± standard deviation.
(PDF)

S5 Fig Effect of STAT6 knockdown on the expression level of CXCL11 and CCL5. Human keratinocytes were transfected with STAT6 siRNA (siSTAT6), control siRNA (siCon), or transfection reagent alone (Mock) before IFNα stimulation for 24 hours. (A) Protein extracts were isolated from the cells and the expression of STAT6 analyzed by western blotting (n = 3). (B and C) The culture medium was isolated and the protein level of (B) CXCL11 and (C) CCL5 was analyzed by ELISA (n = 3).
(PDF)
