# Supplementary figures and images for: STAT2 is involved in the pathogenesis of psoriasis by promoting CXCL11 and CCL5 production by keratinocytes
Source: PLoS One. 2017 May 4;12(5):e0176994. doi: 10.1371/journal.pone.0176994 (PMC5417613; doi:10.1371/journal.pone.0176994)

S1 Fig

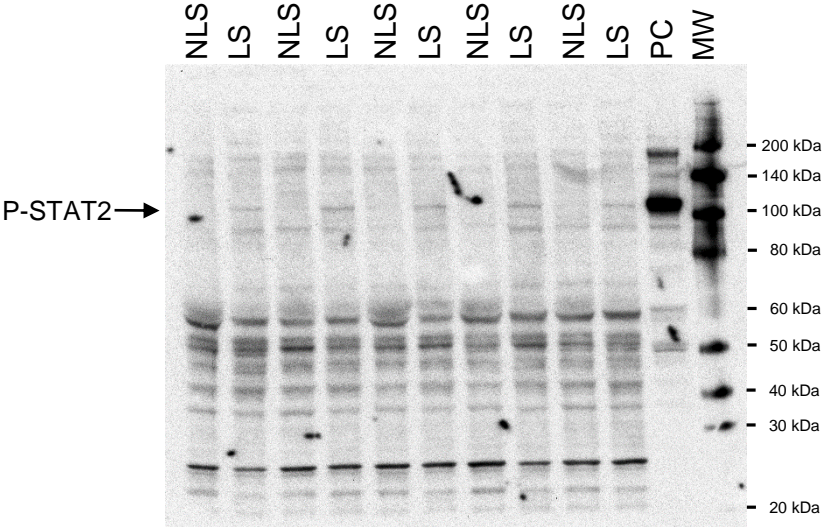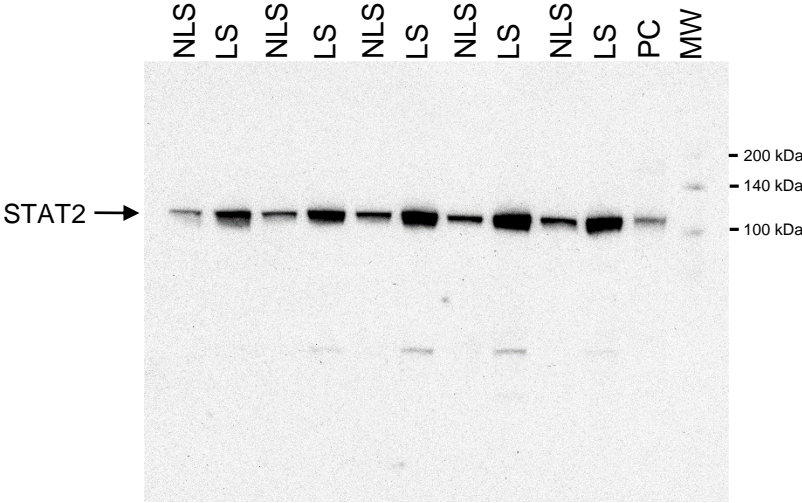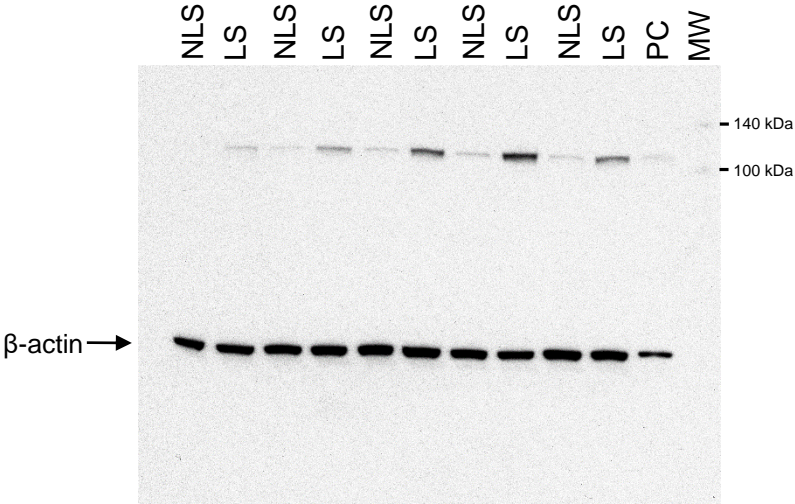

Supplement: S1 Fig — Full-length western blots of the data presented in Fig 1B. (PDF) [file pone.0176994.s001.pdf]

S2 Fig

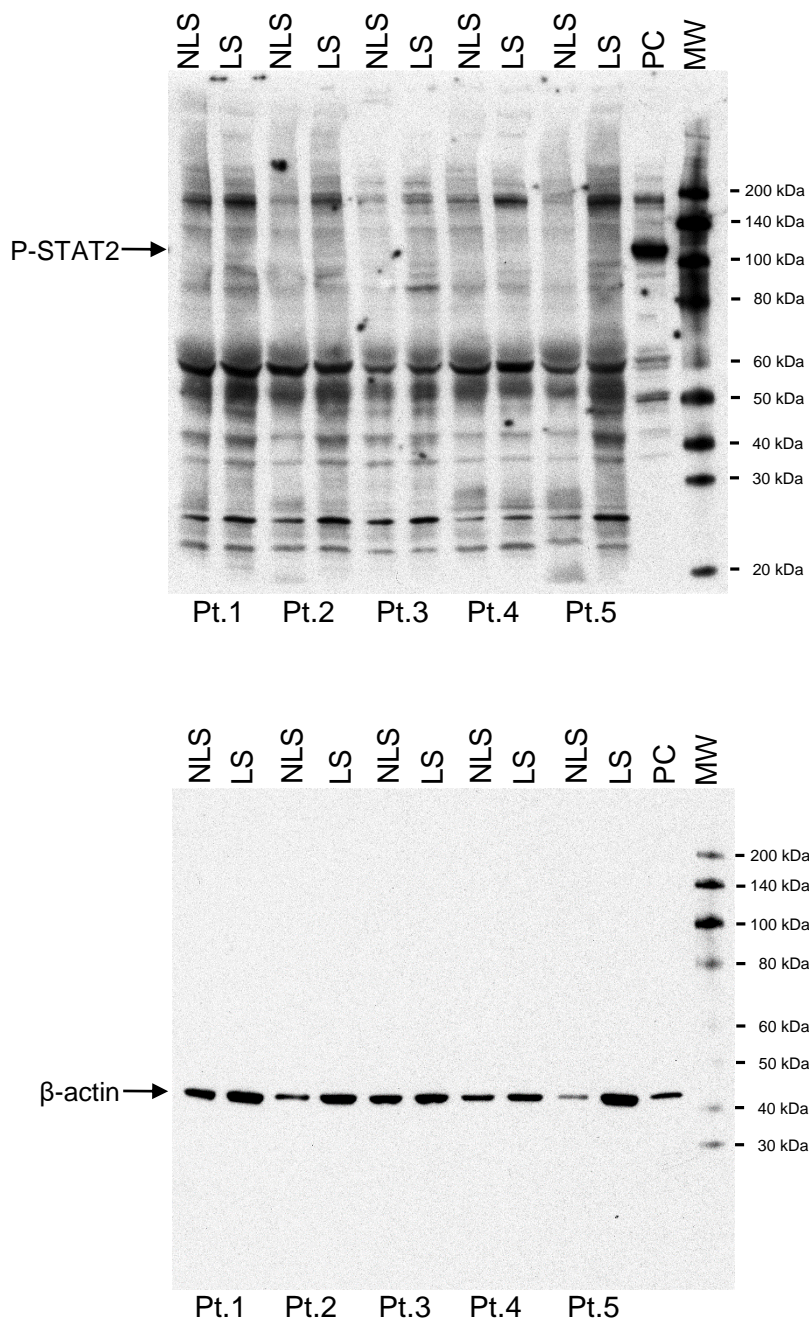

Supplement: S2 Fig — Whole cell protein extracts were prepared from paired keratome biopsies taken from nonlesional (NLS) and lesional (LS) skin from five patients with atopic dermatitis. Phosphorylated STAT2 was analyzed by western blotting. Equal protein loading was assessed by detecting the protein level of β-actin. Protein extract from keratinocytes stimulated with IFNα for 1 hour was included as a positive control (PC). MW; molecular weight marker. (PDF) [file pone.0176994.s002.pdf]

S3 Fig

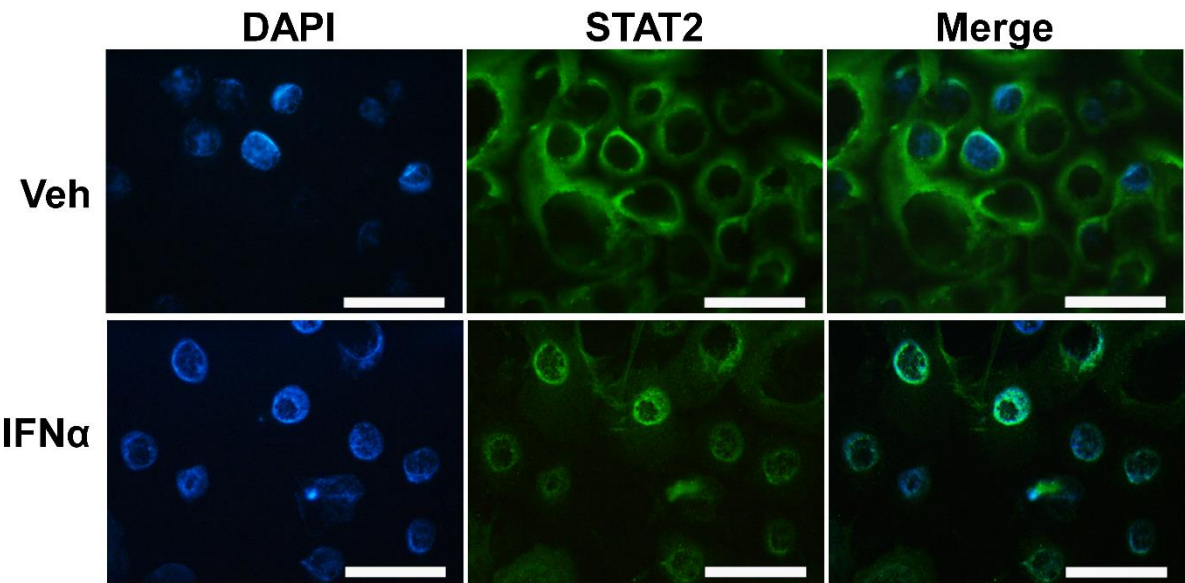

Supplement: S3 Fig — Human keratinocytes were stimulated with or without IFNα (1000 U/ml) for 1 hour. Then the cells were fixed in methanol before immunostaining was performed using an antibody directed against STAT2. Nuclear staining was performed using 4’, 6-diamidine-2’-phenylindole dihydrochloride (DAPI). Green color (Alexa Fluor 488) represents STAT2 protein. Scale bar = 50 μm. (PDF) [file pone.0176994.s003.pdf]

S4 Fig

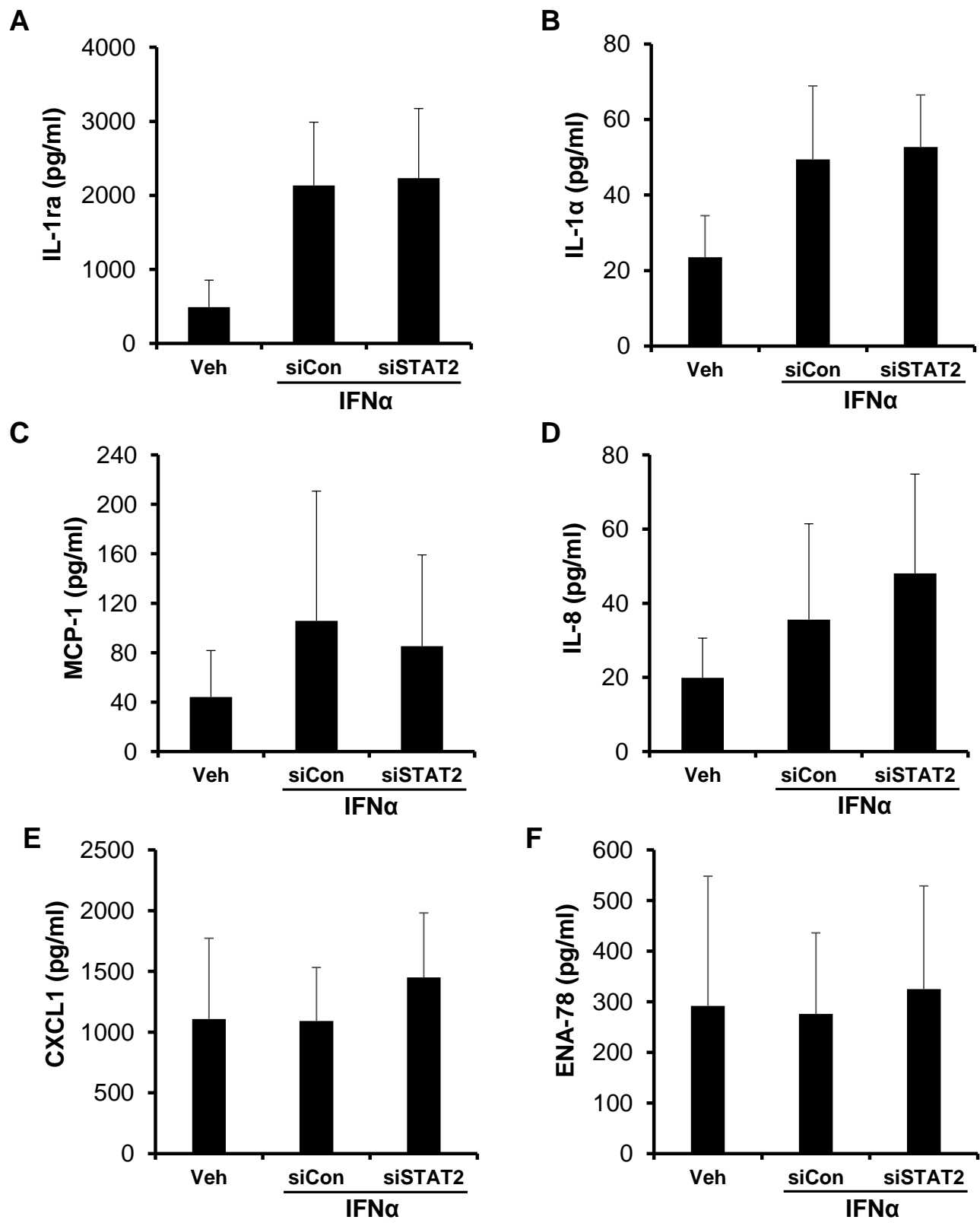

Supplement: S4 Fig — Cultured human keratinocytes were transfected with STAT2 siRNA (siSTAT2) or control siRNA(siCon) before IFNα stimulation for 24 hours. Then the cell culture medium was analyzed for the protein level of (A) IL-1ra, (B) IL-1α, (C) MCP-1, (D) IL-8, (E) CXCL1, and (F) ENA-78 by ELISA (n = 3). Results are expressed as mean ± standard deviation. (PDF) [file pone.0176994.s004.pdf]

S5 Fig

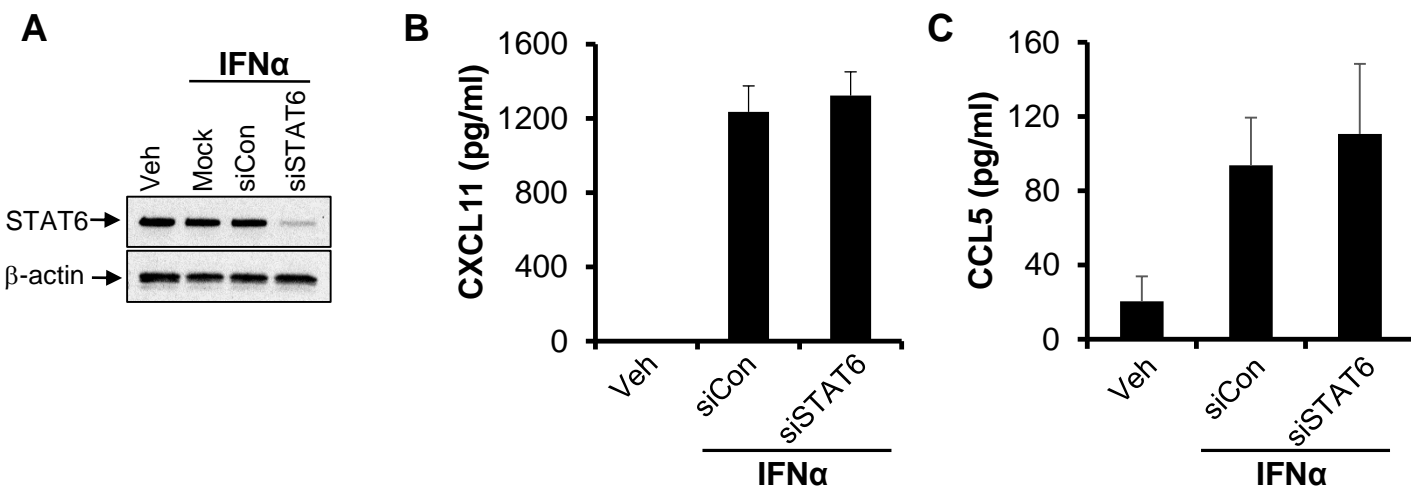

Supplement: S5 Fig — Human keratinocytes were transfected with STAT6 siRNA (siSTAT6), control siRNA (siCon), or transfection reagent alone (Mock) before IFNα stimulation for 24 hours. (A) Protein extracts were isolated from the cells and the expression of STAT6 analyzed by western blotting (n = 3). (B and C) The culture medium was isolated and the protein level of (B) CXCL11 and (C) CCL5 was analyzed by ELISA (n = 3). (PDF) [file pone.0176994.s005.pdf]
